# 2-(1,3-Thia­zol-4-yl)benzimidazolium nitrate monohydrate

**DOI:** 10.1107/S1600536810008433

**Published:** 2010-03-13

**Authors:** Marcos Flores-Alamo, Sandra González-Martínez, Silvia E. Castillo-Blum

**Affiliations:** a Facultad de Química, Universidad Nacional Autónoma de México, 04510 México DF, México

## Abstract

In the title compound, C_10_H_8_N_3_S^+^·NO_3_^−^·H_2_O, one of the N atoms of the benzimidazole unit is protonated, unlike than that in the thia­zole group. This protonation leads to equalization of the bond angles at the two N atoms of the benzimidazole group. The benzimidazole and thia­zole systems are almost coplanar, forming a dihedral angle of 0.5 (2)°. In the crystal, the nitrate anion and water mol­ecule bridge the thia­bendazolium cations through N—H⋯O and O—H⋯O hydrogen bonds, leading to a supra­molecular network based on an infinite one-dimensional chain using [001] as base vector.

## Related literature

For the anti­viral action and anthelmintic activity of substituted benzimidazoles, see: Goodgame *et al.* (1985 ▶). Related structures have been reported: thia­bendazole (Trus & Marsh, 1973 ▶); thia­bendazolium nitrate (Murugesan *et al.*, 1998 ▶; Devereux *et al.*, 2004 ▶); thia­bendazolium perchlorate (Stanley *et al.*, 2002 ▶); thia­bendazolium halide dihydrates (Prabakaran *et al.*, 2000 ▶). For structures of transition metal complexes bearing thia­bendazole as ligand, see: Kowala & Wunderlich (1973 ▶); Udupa & Krebs (1979 ▶); Rong *et al.* (1991 ▶).
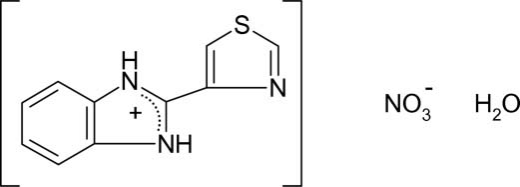


## Experimental

### 

#### Crystal data

C_10_H_8_N_3_S^+^·NO_3_^−^·H_2_O
*M* *_r_* = 282.28
Monoclinic, 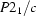

*a* = 7.6140 (3) Å
*b* = 16.3130 (5) Å
*c* = 10.0990 (3) Å
β = 102.731 (4)°
*V* = 1223.53 (7) Å^3^
*Z* = 4
Mo *K*α radiation
μ = 0.28 mm^−1^
*T* = 298 K
0.54 × 0.39 × 0.26 mm

#### Data collection

Oxford Diffraction Xcalibur diffractometer with an Atlas (Gemini Mo) detector
Absorption correction: multi-scan (*CrysAlis RED*; Oxford Diffraction, 2009 ▶) *T*_min_ = 0.920, *T*_max_ = 0.952
5590 measured reflections
2426 independent reflections
1859 reflections with *I* > 2σ(*I*)
*R*_int_ = 0.017

#### Refinement

*R*[*F* ^2^ > 2σ(*F* ^2^)] = 0.036
*wR*(*F* ^2^) = 0.100
*S* = 1.08
2426 reflections
184 parameters
H atoms treated by a mixture of independent and constrained refinement
Δρ_max_ = 0.23 e Å^−3^
Δρ_min_ = −0.21 e Å^−3^

### 

Data collection: *CrysAlis CCD* (Oxford Diffraction, 2009 ▶); cell refinement: *CrysAlis RED* (Oxford Diffraction, 2009 ▶); data reduction: *CrysAlis RED*; program(s) used to solve structure: *SHELXS97* (Sheldrick, 2008 ▶); program(s) used to refine structure: *SHELXL97* (Sheldrick, 2008 ▶); molecular graphics: *ORTEP-3 for Windows* (Farrugia, 1997 ▶); software used to prepare material for publication: *WinGX* (Farrugia, 1999 ▶).

## Supplementary Material

Crystal structure: contains datablocks I, global. DOI: 10.1107/S1600536810008433/bh2274sup1.cif

Structure factors: contains datablocks I. DOI: 10.1107/S1600536810008433/bh2274Isup2.hkl

Additional supplementary materials:  crystallographic information; 3D view; checkCIF report

## Figures and Tables

**Table 1:** Hydrogen-bond geometry (Å, °)

*D*—H⋯*A*	*D*—H	H⋯*A*	*D*⋯*A*	*D*—H⋯*A*
O1*W*—H2*D*⋯O17	0.85 (2)	2.06 (2)	2.903 (2)	168 (2)
O1*W*—H1*D*⋯O16^i^	0.78 (2)	2.24 (3)	2.969 (2)	156 (2)
N1—H1*N*⋯O1*W*^i^	0.85 (2)	1.91 (2)	2.748 (2)	168.5 (18)
N3—H3*N*⋯O16^ii^	0.82 (2)	2.58 (2)	3.300 (2)	147.5 (18)
N3—H3*N*⋯O17^ii^	0.82 (2)	2.02 (2)	2.791 (2)	157 (2)

Symmetry codes: (i) 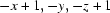; (ii) 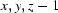.

**Fig. 1.**
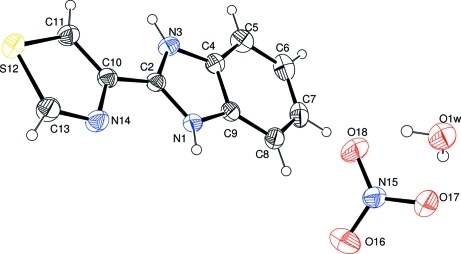
The molecular structure of the title salt, with atom labels and 30% probability displacement ellipsoids for non-H atoms.

## supplementary crystallographic information

### Comment

Substituted benzimidazoles show antiviral action and anthelmintic activity. This has been attributed to their metal-chelating ability (Goodgame *et al.*, 1985). Thiabendazole is a broad-spectrum anthelmintic compound useful in the treatment of human and animal parasitic diseases.

The crystal structures of thiabendazole (Trus & Marsh, 1973), thiabendazolium nitrate (Murugesan *et al.*, 1998; Devereux *et al.*, 2004), thiabendazolium perchlorate (Stanley *et al.*, 2002), thiabendazolium halide dihydrates (Prabakaran *et al.*, 2000), and its complexes with cobalt (Kowala & Wunderlich, 1973), copper (Udupa & Krebs, 1979) and platinum (Rong *et al.*, 1991) have been reported. The present paper deals with the crystal structure of a protonated thiabendazole moiety, namely, thiabendazolium nitrate hydrate.

The asymmetric unit of the title salt contains one protonated 2-(4-thiazolyl)-1*H*-benzimidazol-1-ium cation, one nitrate anion and one water molecule, shown in Fig. 1. The cation is protonated on the benzimidazole iminic nitrogen atom, resulting in delocalization of the double bond over the N—C—N fragment, with C—N distances of 1.326 (2) and 1.327 (2) Å (Table 1), in contrast to the benzimidazole group in the crystal structure of free thiabendazole, where the two bond lengths are different (Trus & Marsh, 1973).

The C—C bond connecting the two ring systems has a length of 1.445 (2) Å, which is the same bond length, within experimental error, as that in neutral thiabendazole. This value suggests appreciable delocalization across this bond (Prabakaran *et al.*, 2000).

The benzimidazole and thiazole systems are coplanar, the dihedral angle between them is 0.5 (2)°.

The thiabendazole cation is involved in a pair of N—H···O, O—H···O hydrogen bonds [N1···O1w: 2.748 (2) Å and N3···O17: 2.791 (2) Å], while the nitrate anion and water molecule display hydrogen bonding (Table 2), which lead to an infinite one-dimensional chain with base vector [0 0 1].

### Experimental

The reaction mixture of 2-(4-thiazolyl)benzimidazole (0.3686 g, 1.83 mmol) with [Fe(DMSO)_6_]NO_3_ (0.3549 g, 0.5 mmol) in acetonitrile (60 ml) was refluxed for 10 h. It yielded pale-yellow crystals of (C_10_H_8_N_3_S)(NO_3_).H_2_O as a byproduct when the solution was left to stand at room temperature for a couple of days.

### Refinement

H atoms bonded to N and O atoms were located in difference maps and were refined with free coordinates and *U*_iso_(H) = 1.5*U*_eq_(N) and 1.2*U*_eq_(O). H atoms attached to C atoms were placed in geometrically idealized positions and refined as riding on their parent atoms, with C—H = 0.93 Å and *U*_iso_(H) = 1.2*U*_eq_(C).

### Crystal data

**Table:**

C_10_H_8_N_3_S^+^·NO_3_^−^·H_2_O	*F*(000) = 584
*M**_r_* = 282.28	*D*_x_ = 1.532 Mg m^−^^3^
Monoclinic, *P*2_1_/*c*	Mo *K*α radiation, λ = 0.71073 Å
Hall symbol: -P 2ybc	Cell parameters from 3277 reflections
*a* = 7.6140 (3) Å	θ = 3.2–26.0°
*b* = 16.3130 (5) Å	µ = 0.28 mm^−^^1^
*c* = 10.0990 (3) Å	*T* = 298 K
β = 102.731 (4)°	Prism, pale yellow
*V* = 1223.53 (7) Å^3^	0.54 × 0.39 × 0.26 mm
*Z* = 4	

### Data collection

**Table:**

Oxford Diffraction Xcalibur diffractometer with an Atlas (Gemini Mo) detector	2426 independent reflections
Radiation source: X-ray	1859 reflections with *I* > 2σ(*I*)
graphite	*R*_int_ = 0.017
Detector resolution: 10.4685 pixels mm^-1^	θ_max_ = 26.1°, θ_min_ = 3.2°
ω scans	*h* = −9→7
Absorption correction: multi-scan (*CrysAlis RED*; Oxford Diffraction, 2009)	*k* = −19→20
*T*_min_ = 0.920, *T*_max_ = 0.952	*l* = −12→10
5590 measured reflections	

### Refinement

**Table:**

Refinement on *F*^2^	Primary atom site location: structure-invariant direct methods
Least-squares matrix: full	Secondary atom site location: difference Fourier map
*R*[*F*^2^ > 2σ(*F*^2^)] = 0.036	Hydrogen site location: inferred from neighbouring sites
*wR*(*F*^2^) = 0.100	H atoms treated by a mixture of independent and constrained refinement
*S* = 1.08	*w* = 1/[σ^2^(*F*_o_^2^) + (0.0565*P*)^2^ + 0.057*P*] where *P* = (*F*_o_^2^ + 2*F*_c_^2^)/3
2426 reflections	(Δ/σ)_max_ < 0.001
184 parameters	Δρ_max_ = 0.23 e Å^−^^3^
0 restraints	Δρ_min_ = −0.21 e Å^−^^3^
0 constraints	

### Fractional atomic coordinates and isotropic or equivalent isotropic displacement parameters (Å^2^)

**Table:**

	*x*	*y*	*z*	*U*_iso_*/*U*_eq_	
C2	0.7417 (2)	−0.01688 (10)	0.01259 (16)	0.0385 (4)	
C4	0.8456 (2)	0.11013 (10)	0.02130 (18)	0.0416 (4)	
C5	0.8891 (3)	0.18937 (11)	−0.0082 (2)	0.0542 (5)	
H5	0.8578	0.2102	−0.096	0.065*	
C6	0.9813 (3)	0.23603 (12)	0.0988 (2)	0.0606 (5)	
H6	1.0132	0.2897	0.084	0.073*	
C7	1.0266 (2)	0.20260 (12)	0.2293 (2)	0.0576 (5)	
H7	1.0923	0.2346	0.2992	0.069*	
C8	0.9784 (2)	0.12463 (12)	0.25917 (19)	0.0517 (5)	
H8	1.0062	0.1042	0.3473	0.062*	
C9	0.8865 (2)	0.07819 (10)	0.15118 (16)	0.0416 (4)	
C10	0.6544 (2)	−0.09278 (9)	−0.03838 (16)	0.0387 (4)	
C11	0.5761 (2)	−0.10865 (11)	−0.17031 (17)	0.0467 (4)	
H11	0.5695	−0.0718	−0.2415	0.056*	
C13	0.5667 (3)	−0.21780 (11)	−0.01579 (18)	0.0507 (4)	
H13	0.5495	−0.2667	0.027	0.061*	
N1	0.81909 (19)	−0.00164 (8)	0.14122 (14)	0.0403 (3)	
N3	0.7559 (2)	0.04857 (9)	−0.06229 (15)	0.0434 (4)	
N14	0.6484 (2)	−0.15582 (9)	0.05111 (15)	0.0487 (4)	
N15	0.6705 (2)	0.07673 (9)	0.58671 (15)	0.0502 (4)	
O1W	0.2195 (2)	0.07887 (10)	0.62504 (15)	0.0621 (4)	
O16	0.7817 (3)	0.02340 (12)	0.61835 (19)	0.1061 (7)	
O17	0.6049 (2)	0.10779 (9)	0.67836 (14)	0.0724 (4)	
O18	0.6223 (3)	0.09795 (10)	0.47009 (14)	0.0989 (6)	
S12	0.49270 (6)	−0.20485 (3)	−0.18646 (4)	0.05076 (18)	
H2D	0.329 (3)	0.0937 (14)	0.647 (2)	0.076*	
H1D	0.205 (3)	0.0427 (15)	0.574 (2)	0.076*	
H1N	0.814 (3)	−0.0313 (12)	0.210 (2)	0.061*	
H3N	0.722 (3)	0.0536 (13)	−0.145 (2)	0.061*	

### Atomic displacement parameters (Å^2^)

**Table:**

	*U*^11^	*U*^22^	*U*^33^	*U*^12^	*U*^13^	*U*^23^
C2	0.0382 (9)	0.0415 (9)	0.0363 (9)	0.0061 (7)	0.0094 (7)	0.0024 (7)
C4	0.0385 (9)	0.0433 (9)	0.0447 (9)	−0.0003 (7)	0.0131 (7)	0.0001 (7)
C5	0.0536 (11)	0.0513 (11)	0.0609 (12)	−0.0058 (8)	0.0193 (10)	0.0072 (9)
C6	0.0486 (11)	0.0507 (11)	0.0877 (16)	−0.0080 (9)	0.0261 (11)	−0.0116 (11)
C7	0.0421 (10)	0.0577 (12)	0.0726 (13)	−0.0018 (8)	0.0119 (9)	−0.0247 (10)
C8	0.0462 (10)	0.0564 (11)	0.0496 (10)	0.0067 (8)	0.0041 (8)	−0.0116 (9)
C9	0.0379 (9)	0.0435 (9)	0.0437 (9)	0.0060 (7)	0.0100 (7)	−0.0020 (8)
C10	0.0369 (9)	0.0413 (9)	0.0385 (9)	0.0043 (7)	0.0098 (7)	0.0007 (7)
C11	0.0543 (11)	0.0477 (10)	0.0371 (9)	0.0004 (8)	0.0080 (8)	0.0035 (7)
C13	0.0614 (11)	0.0457 (10)	0.0452 (10)	−0.0069 (8)	0.0124 (9)	0.0037 (8)
N1	0.0464 (8)	0.0403 (8)	0.0337 (7)	0.0064 (6)	0.0076 (6)	0.0026 (6)
N3	0.0473 (9)	0.0488 (8)	0.0340 (7)	−0.0021 (6)	0.0086 (7)	0.0057 (7)
N14	0.0591 (9)	0.0464 (8)	0.0390 (8)	−0.0046 (7)	0.0070 (7)	0.0044 (7)
N15	0.0613 (10)	0.0447 (8)	0.0414 (8)	0.0024 (7)	0.0044 (7)	0.0033 (7)
O1W	0.0733 (10)	0.0623 (9)	0.0512 (8)	0.0057 (8)	0.0149 (8)	0.0038 (6)
O16	0.1230 (16)	0.1025 (14)	0.0874 (12)	0.0633 (12)	0.0118 (11)	0.0130 (10)
O17	0.0828 (11)	0.0859 (11)	0.0469 (8)	0.0184 (8)	0.0104 (7)	−0.0106 (7)
O18	0.1616 (18)	0.0918 (12)	0.0357 (8)	0.0366 (11)	0.0057 (10)	0.0079 (7)
S12	0.0541 (3)	0.0533 (3)	0.0437 (3)	−0.0063 (2)	0.0082 (2)	−0.0065 (2)

### Geometric parameters (Å, °)

**Table:**

N1—C2	1.326 (2)	C8—C9	1.385 (2)
N1—C9	1.395 (2)	C8—H8	0.93
N3—C2	1.327 (2)	C10—C11	1.359 (2)
N3—C4	1.390 (2)	C11—S12	1.6874 (18)
N14—C10	1.376 (2)	C11—H11	0.93
N14—C13	1.296 (2)	C13—S12	1.7045 (19)
C2—C10	1.445 (2)	C13—H13	0.93
C4—C9	1.382 (2)	N1—H1N	0.85 (2)
C4—C5	1.383 (2)	N3—H3N	0.82 (2)
C5—C6	1.380 (3)	N15—O18	1.205 (2)
C5—H5	0.93	N15—O16	1.207 (2)
C6—C7	1.398 (3)	N15—O17	1.2516 (19)
C6—H6	0.93	O1W—H2D	0.85 (2)
C7—C8	1.375 (3)	O1W—H1D	0.78 (2)
C7—H7	0.93		
			
C2—N1—C9	108.82 (13)	C7—C8—H8	121.7
C2—N1—H1N	126.9 (14)	C9—C8—H8	121.7
C9—N1—H1N	123.4 (13)	C4—C9—C8	120.69 (16)
C2—N3—C4	109.03 (14)	C4—C9—N1	106.34 (14)
C2—N3—H3N	127.8 (15)	C8—C9—N1	132.97 (16)
C4—N3—H3N	123.2 (15)	C11—C10—N14	115.50 (15)
N1—C2—N3	109.45 (15)	C11—C10—C2	125.47 (15)
N1—C2—C10	125.43 (14)	N14—C10—C2	119.03 (14)
N3—C2—C10	125.13 (15)	C10—C11—S12	110.26 (13)
C9—C4—C5	122.87 (17)	C10—C11—H11	124.9
C9—C4—N3	106.36 (14)	S12—C11—H11	124.9
C5—C4—N3	130.76 (16)	N14—C13—S12	116.36 (14)
C6—C5—C4	116.81 (19)	N14—C13—H13	121.8
C6—C5—H5	121.6	S12—C13—H13	121.8
C4—C5—H5	121.6	C13—N14—C10	108.79 (15)
C5—C6—C7	120.04 (18)	O18—N15—O16	120.71 (18)
C5—C6—H6	120	O18—N15—O17	121.43 (17)
C7—C6—H6	120	O16—N15—O17	117.84 (16)
C8—C7—C6	123.02 (19)	O18—N15—O17	121.43 (17)
C8—C7—H7	118.5	O16—N15—O17	117.84 (16)
C6—C7—H7	118.5	H2D—O1W—H1D	112 (2)
C7—C8—C9	116.52 (18)	C11—S12—C13	89.08 (8)
			
C9—C4—C5—C6	1.6 (3)	N14—C10—C11—S12	−0.39 (19)
N3—C4—C5—C6	−179.56 (17)	C2—C10—C11—S12	179.16 (13)
C4—C5—C6—C7	0.2 (3)	N3—C2—N1—C9	0.41 (18)
C5—C6—C7—C8	−2.2 (3)	C10—C2—N1—C9	−179.28 (14)
C6—C7—C8—C9	2.3 (3)	C4—C9—N1—C2	−0.09 (17)
C5—C4—C9—C8	−1.5 (3)	C8—C9—N1—C2	−179.72 (17)
N3—C4—C9—C8	179.44 (14)	N1—C2—N3—C4	−0.58 (18)
C5—C4—C9—N1	178.82 (15)	C10—C2—N3—C4	179.11 (14)
N3—C4—C9—N1	−0.25 (17)	C9—C4—N3—C2	0.51 (18)
C7—C8—C9—C4	−0.5 (2)	C5—C4—N3—C2	−178.47 (18)
C7—C8—C9—N1	179.10 (16)	S12—C13—N14—C10	−0.2 (2)
N1—C2—C10—C11	−179.51 (16)	C11—C10—N14—C13	0.4 (2)
N3—C2—C10—C11	0.8 (3)	C2—C10—N14—C13	−179.21 (15)
N1—C2—C10—N14	0.0 (2)	C10—C11—S12—C13	0.22 (14)
N3—C2—C10—N14	−179.62 (15)	N14—C13—S12—C11	−0.01 (15)

### Hydrogen-bond geometry (Å, °)

**Table:**

*D*—H···*A*	*D*—H	H···*A*	*D*···*A*	*D*—H···*A*
O1W—H2D···O17	0.85 (2)	2.06 (2)	2.903 (2)	168 (2)
O1W—H1D···O16^i^	0.78 (2)	2.24 (3)	2.969 (2)	156 (2)
N1—H1N···O1W^i^	0.85 (2)	1.91 (2)	2.748 (2)	168.5 (18)
N3—H3N···O16^ii^	0.82 (2)	2.58 (2)	3.300 (2)	147.5 (18)
N3—H3N···O17^ii^	0.82 (2)	2.02 (2)	2.791 (2)	157 (2)

Symmetry codes:  (i) −*x*+1, −*y*, −*z*+1; (ii) *x*, *y*, *z*−1.
